# Stage-specific transcriptome of *Bursaphelenchus xylophilus* reveals temporal regulation of effector genes and roles of the dauer-like stages in the lifecycle

**DOI:** 10.1038/s41598-019-42570-7

**Published:** 2019-04-15

**Authors:** Suguru E. Tanaka, Mehmet Dayi, Yasunobu Maeda, Isheng J. Tsai, Ryusei Tanaka, Mark Bligh, Yuko Takeuchi-Kaneko, Kenji Fukuda, Natsumi Kanzaki, Taisei Kikuchi

**Affiliations:** 1 Laboratory of Forest Botany, Graduate School of Agricultural and Life Sciences, the University of Tokyo, Tokyo, 113-8657 Japan; 2 Division of Parasitology, Faculty of Medicine, University of Miyazaki, Miyazaki, 889-1692 Japan; 3 Forestry Vocational School, Duzce University, 81620 Duzce, Turkey; 4 Biodiversity Research Center, Academia Sinica, Taipei, Taiwan; 5 Laboratory of Terrestrial Microbial Ecology, Graduate School of Agriculture, Kyoto University, Kyoto, 606-8502 Japan; 6 Kansai Research Center, Forestry and Forest Products Research Institute, Kyoto, 612-0855 Japan

## Abstract

The pine wood nematode *Bursaphelenchus xylophilus* is the causal agent of pine wilt disease, one of the most devastating forest diseases in East Asian and West European countries. The lifecycle of *B. xylophilus* includes four propagative larval stages and gonochoristic adults which are involved in the pathogenicity, and two stages of dispersal larvae involved in the spread of the disease. To elucidate the ecological roles of each developmental stage in the pathogenic life cycle, we performed a comprehensive transcriptome analysis using RNA-seq generated from all developmental stages of *B. xylophilus* and compared transcriptomes between stages. We found more than 9000 genes are differentially expressed in at least one stage of the life cycle including genes involved in general nematode biology such as reproduction and moulting but also effector genes likely to be involved in parasitism. The dispersal-stage transcriptome revealed its analogy to *C. elegans* dauer and the distinct roles of the two larval stages from each other regarding survival and transmission. This study provides important insights and resources to understand *B. xylophilus* parasitic biology.

## Introduction

**Figure 1:**
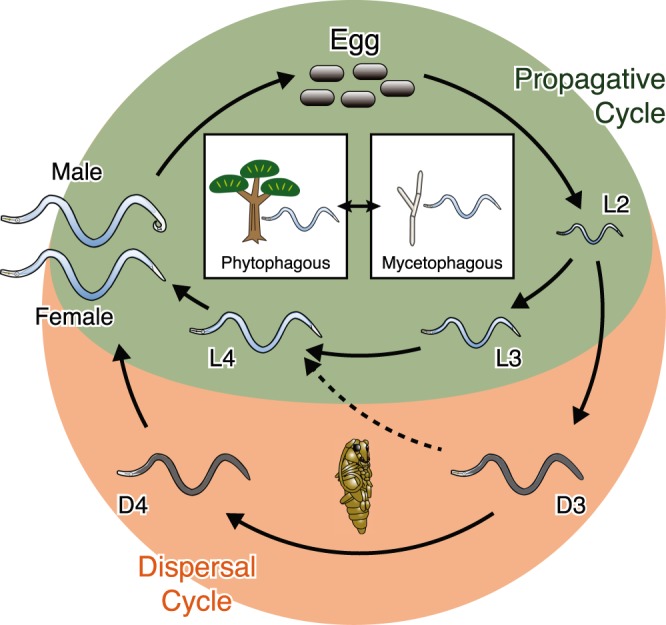
Life cycle of *B. xylophilus*. In favourable conditions *B. xylophilus* multiplies via a propagative cycle by feeding on plant cell (phytophagous phase) or fungal hyphae (mycetophagous phase). In unfavourable conditions, the nematode switches to the dispersal cycle in which the second-stage larvae (L2) moult to the dispersal third-stage juvenile (D3) and subsequently to the dispersal fourth-stage juvenile (D4) in a presence of the vector insect, which enters the vector beetle for dispersion.

The pine wood nematode, *Bursaphelenchus xylophilus*, is the causal agent of Pine Wilt Disease (PWD), one of the most serious forest diseases in East Asian and West European countries^1^. This migratory endoparasitic nematode has a complex lifecycle which includes two distinct dietary phases (phytophagous and mycetophagous phases) and two developmental forms (propagative and dispersal forms) (Fig. 1). In the life cycle, the nematodes enter the host pine tree when the vector beetle (*Monochamus* spp.), which has a phoretic association with *B. xylophilus*, undergoes maturation feeding. On entering the tree the nematodes feed on plant epithelial cells around the cortical and xylem tissues^2^, spreading throughout the tree by migrating through the tissues (phytophagous phase), leading to wilting symptoms that result in the death of the tree within a year of infection. After the tree becomes symptomatic, the nematodes start feeding on fungi that invade the tree and multiply into millions (mycetophagous phase). In both phases, *B. xylophilus* develops from an egg to an adult via three propagative larvae (L2, L3 and L4. Nota bene: L1 moults to L2 inside the egg). However, when conditions become harsh, *B. xylophilus* enters into its dispersal phase in which L2 moults to an alternative L3 stage called the third stage dispersal juvenile (D3). Furthermore, in the presence of the vector beetle D3 moults to an alternative fourth stage larva called the fourth stage dispersal juvenile (D4). The D4 nematodes are transmitted by the vector to new healthy host trees to complete the pathogenic life cycle^3,4^ (Fig. 1).

The dispersal stages of *B. xylophilus* are distinguishable by their morphology^5–7^, and thought to be analogous to the *C. elegans* arrested stage (dauer)^8,9^. However, whereas *C. elegans* dauer is an alternative form of its third stage larva, *B. xylophilus* has two developmental stages with dauer-like characteristics (D3 and D4). Interestingly, the later stage (D4) is morphologically more similar to the *C. elegans* third-stage dauer whose mouth is closed and body is covered with thick cuticle^10^, whilst D3 exhibit an active feeding behaviour, but also shows high stress tolerance and extended longevity^11,12^. Environmental conditions to form *B. xylophilus* D3 are similar to those of *C. elegans* dauer including scarcity of food and high nematode density^10–12^, whereas *B. xylophilus* D4 forms only with the vector beetle stimulus^13,14^. Because those stages are essential for the transmission of the pathogen, understanding molecular mechanisms underlying formations and maintenance of those stages are important for developing efficient disease control methods.

In contrast, the propagative cycle of *B. xylophilus* contains stages that are responsible for inducing the typical wilting symptom of the host tree. Like other plant parasitic nematodes, *B. xylophilus* produces and secretes a variety of proteins, called effectors, to manipulate their hosts in order to obtain the nutrients required for development and reproduction. Identified *B. xylophilus* effectors include cell wall degrading enzymes and peptidases for feeding and movement within the tree^15–18^, and anti-oxidant and detoxifying enzymes against host defences^19,20^. The regulation of transcription of effector proteins varies depending on the requirements of the life-cycle stage^21^. Recently two independent studies using RNA deep sequencing (RNA-seq) to compare the mycetophagous with phytophagous stages revealed that several effector candidate genes including those encoding peptidases, glycosyl hydrolases and detoxification proteins are differentially regulated during the infection^20,22^, suggesting transcriptional and physiological changes are important for the parasitism of *B. xylophilus*. However, those RNA-seq studies used mixed-stage nematodes and it remains unclear if each stage has any specific roles in the pathogenic life cycle.

In this study, we aimed to obtain comprehensive transcriptional profiles during development of *B. xylophilus* using stage-specific RNA-seq data. To achieve accurate analyses, we produced an improved genome assembly and gene models using newly obtained genome/transcriptome sequencing data^23^. Then, we performed RNA-seq comparisons between developmental stages to understand stage-dependent gene expression pattern. In particular, we generated transcriptome data for the dispersal (dauer-like) stages for the first time as well as the propagative stages and achieved a comprehensive gene expression profile.

## Results

### Reference Genome Improvement

We generated a new genome assembly using sequencing data from multiple Illumina libraries (see Materials and Methods). The new assembly is 75.9 Mb in length, which is 1.3 Mb larger than the previously published version (v1.2), composed of 501 scaffolds with the largest scaffold of 4.1 Mb (Table S1). Mapping ratio of Illumina short reads generated using *B. xylophilus* genomic DNA to the new reference genome was 99.1% whereas 98.3% reads were mapped to the previous version, suggesting a higher genome coverage by the new assembly. We have predicted gene models on the new assembly using RNA-seq alignments as hints (please see Methods). The new gene set (v2.0) contains 16,346 gene models, which is ~2,000 smaller than the previous version (v1.2) but having a longer average gene length with more exons per gene (Table S1). Gene model comparison revealed that the new gene set has a high number of full-length genes, some of which are truncated or fragmented in the previous version, e.g., a new gene model BXYJ_1053000.1 (1107 amino acids) corresponds to three old gene models which are located next to each other on a scaffold (BUX_s00543.22, BUX_s00543.23 and BUX_s00543.24) and matched *C. elegans prp-*6 gene (968 amino acids) (Fig. S1).

### RNA preparation and sequencing

RNA was extracted from each nematode developmental stage from the egg to the adult (egg, L2, L3, L4, adult female and adult male). RNA samples were also prepared from the two dauer-like stages (D3 and D4). At least two biological replicates of at least 20 million mRNA-seq reads in each stage was produced (except one D4 sample (D4_rep1) with ~10 million reads) (Table S2). Aligning the RNA-seq reads to the reference genome exhibited a high mapping ratio for most of the samples (87.3% to 92.9%), suggesting high quality RNA-seq data. D4 samples were the exceptions having relatively low mapping ratios (43.3% to 75.4%) (Table S2) partly due to mRNA contamination from the insects from which the nematodes were isolated, and some nematode rRNA contamination (Table S2). Overall, in each stage, 12301–14699 genes had >5 mapped reads; adult female was the largest (14699 genes having >5 mapped reads) and D4 the smallest (12301 genes having >5 mapped reads) (Fig. S2).

**Figure 2:**
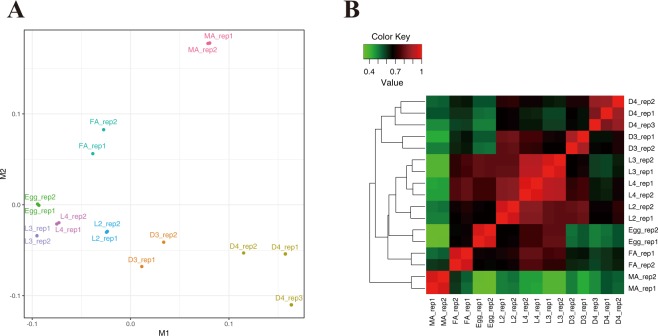
Multi-dimensional scaling plot (**A**) and heat map of clustered correlation matrix (**B**) of all RNA-seq samples. L2, L3 and L4: propagative second, third and fourth stage, respectively, MA: male, FA: female, D3 and D4: dispersal third and fourth stage, respectively.

Gene expression values (read count and FPKM (Fragments Per Kilobase of transcript per Million mapped reads)) were calculated for each gene model and used for further comparisons (Table S2). Multidimensional scaling (MDS) analysis of FPKM showed that replicates from the same stages are similar to each other (Fig. 2A). The propagative stages (L2, L3 and L4) and egg stage were positioned closely to each other whereas adult female, adult male, D3 and D4 samples were distantly located from all other samples with relatively high deviations within the replicates (except adult male). Consistently, high correlation was observed between propagative larval stages (L2, L3 and L4) with the highest Pearson’s correlation coefficient of 0.92 between L3 and L4 (Fig. 2B). Correlations between the adult male and other stages were relatively low (0.26 to 0.43). Two dispersal stages (D3 and D4) formed a heat map cluster, however D3 showed higher correlation values with L2 than D4 (Fig. 2B). Both in the MDS plot and the heat map, the egg stage is located between the larval stage cluster (L2, L3 and L4) and adult female. This is probably because the gravid adult females contain many eggs inside the body and the egg samples contained a range of developmental stages from the early immediate post-delivery stage to later stages containing L1 larvae.

### Changes in gene expression during development

Comparing gene expression values between all possible stage pairs identified a total of 9612 genes that were differentially expressed in any pairs with a minimum fold change of 2 and a FDR of <0.001 (Table S3). The number of DE genes was smallest at L3-L4 comparison (347) and largest at egg-male comparison (4327) (Table S3).

**Figure 3:**
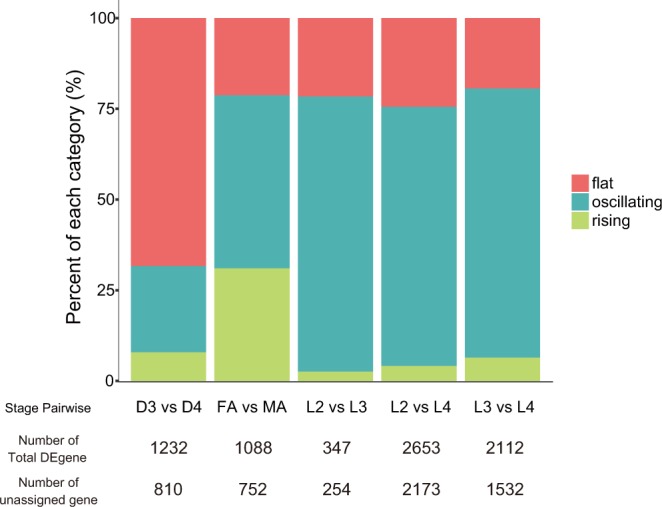
Proportions of differentially expressed genes assigned to “flat”, “rising” and “oscillating” genes in pair-wise comparisons. The “flat” category is higher in the two dauer-like stage comparison (D3-D4) and “rising” has higher proportions in adult female and male comparison (FA-MA) than the other pairs, whereas the “oscillating” gene category are dominant in comparisons between larvae (L2-L3, L2-L4 and L3-L4).

Pairwise comparisons between propagative larvae (L2, L3 and L4) showed smaller numbers (347 to 1232) of DE genes than other pairwise combinations (Table S3). Those DE genes were enriched in GO (Gene Ontology) terms of structural constituent of cuticle (GO:0042302), collagen (GO:0005581) and lipid binding (GO:0008289) (Table S4) and contain many orthologues of the *C. elegans* “oscillating genes”, which have oscillatory expressions within a developmental stage and mainly involve moulting functions, constituting 19.9–26.0% of the total DE genes (65.7–73.2% excluding ‘unclassified’) (Fig. 3 and Table S5). In contrast, non-DE genes in the analysis were mainly composed (45.3% of the total, 51.3% excluding ‘unclassified’) of orthologues of *C. elegans* ‘flat genes’ (Table S5). Other than oscillating genes, genes having GO terms of proteolysis (GO:0006508) are up-regulated in L3 and L4 compared to L2 (Fig. 3 and Table S4).

**Figure 4:**
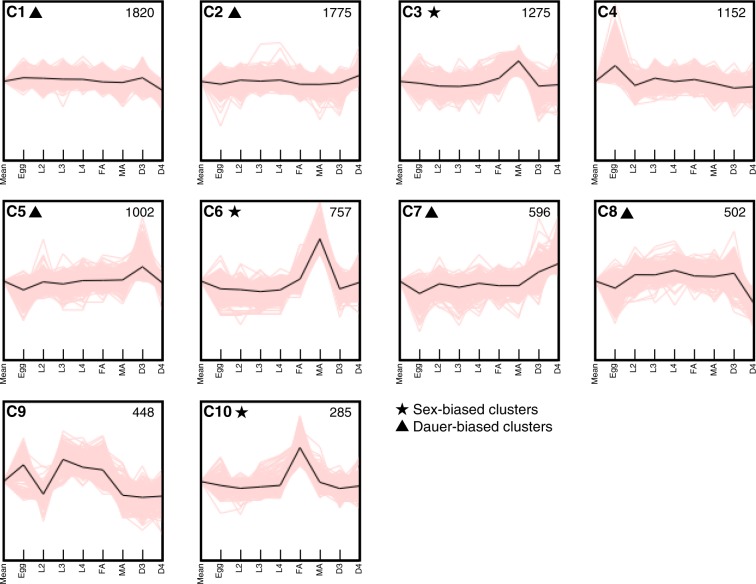
Clustering analysis of genes differentially expressed between developmental stages. A total of 9612 genes are clustered into 10 (C1 to C10) based on expression dynamics by K-means clustering. The upper-left alphanumeric values designate the number of the cluster. Sex-biased and dauer-biased clusters were indicated by stars and triangles, respectively. The upper-right values in each cluster shows cluster size (number of genes). Black lines indicate representative transcriptional expressions.

Expression patterns of the 9612 DE genes along the development can be grouped into 10 clusters (C1 to C10) using the k-means method (Fig. 4) with cluster sizes ranging from 285 to 1820 genes. These clusters can be further classified roughly into sex-biased (C3, C6 and C10 clusters), dauer-biased (C1, C2, C5, C7 and C8 clusters) and others (C4 and C9 clusters) (Fig. 4).

Cluster C4 genes showed high expression in the egg stage and relatively flat in other stages, representing genes involved in later embryo development (Fig. 4). Enriched GO terms in these genes contain many categories related to cell cycle (GO:0007049), suggesting higher cell division activity in eggs than other stages (Table S6D).

Expression values of cluster C9 genes are high in egg, L3, L4 and adult female, and low in L2, D3, D4 and adult male stages (Fig. 4). This cluster is enriched with GO terms related to the moulting cycle (GO:0042303). In addition, 166 out of 448 genes in this cluster are orthologues of *C. elegans* ‘oscillating’ genes which show pervasive and phase-locked oscillations of gene expression in propagative larvae, suggesting the cluster C9 genes are changeable depending on developmental stages (Table S6I).

### Sex-biased and germline-enriched transcriptome

Two clusters (C3 and C6) exhibit up-regulated gene expression in the male stage and contained 1275 and 757 genes, respectively. The C6 cluster genes showed a sharper peak in the male stage than C3 genes but both are enriched in similar GO terms including protein kinase activity (GO:0004672), protein phosphorylation (GO:0006468), dephosphorylation (GO:0016311), and ATP binding (GO:0005524) (Fig. 4 and Table S6C,F). In contrast, C10 cluster genes showed high expression in the female stage and are enriched in GO terms involved in proteolysis (carboxy (GO:0004180), cysteine-type (GO:0008234) and serine-type (GO:0008236) peptidases) and structural constituent of cuticle (GO:0042302) (Table S6J).

A direct comparison of the adult female and adult male stages identified 2653 DE genes (Table S3). Within those, 968 and 1685 genes are female-biased and male-biased, respectively. We then directly compared adult female and adult male stage gene expression data to elucidate up-regulated expressions in germline and somatic tissues using the strategy described in Choi *et al*.^24^. Using these criteria (please see Methods), a total of 1977 and 1550 genes were identified as germline-enriched and somatic-enriched genes, respectively (Table S7). GO term categories including DNA binding (GO:0003677), regulation of macromolecule biosynthetic process (GO:0010556) and regulation of RNA biosynthetic process (GO:2001141) are highly represented in germline-enriched genes, whereas genes implicated in proteolysis (GO:0070011), peptidase activity (GO:0008233) and extracellular region (GO:0005576) are more frequently found in the somatic-enriched gene set (Table S7).

### Dispersal stage gene expressions

As contrasted with the dauer stage of *C. elegans*, which is an alternative third stage larva, *B. xylophilus* has two developmental stages with dauer-like characteristics (D3 and D4) (Fig. 1). Heatmap (Fig. 2B) and MDS plot of expression data (Fig. 2A) suggested the two dauer-like stages are transcriptionally very distinct from the propagative stages.

Cluster C1 and C8 genes showed down-regulation at D4 (Fig. 4). C1 contained a large number of genes (1820 genes) and is enriched in a wide variety of GO terms involved in general biological processes (Table S6A,H), suggesting a low metabolic activity of D4 stage. C8 (502 genes) is enriched in GO terms of proteolysis, especially in aspartic-type (GO:0004190 and GO:0070001) and cysteine-type (GO:0008234) peptidases, which are expanded in the *B. xylophilus* genome and involved in feeding behaviour of the nematode^19,23^, reflecting the D4 characteristics of the covered mouth and lack of feeding behaviour. This corresponds to the observation of low expression of C8 genes in the egg stage (Fig. 4).

In contrast, the C2 cluster (1775 genes) is up-regulated at D4 and enriched in signal transduction-related GO terms including potassium channel activity (GO:0015079), neuropeptide receptor activity (GO:0008188), G-protein coupled receptor activity (GO:0004930), and integral component of membrane (GO:0016021) (Table S6B). The C7 cluster (596 genes), up-regulated at both D3 and D4, is enriched in “neuropeptide signalling pathway” GO term (GO:0007218) (Table S6G). The C5 cluster showed higher expression at D3 than D4 (Fig. 4). GO enrichment analysis revealed C5 genes are enriched in the categories involved in peptide metabolic processes (GO:0006518) with more anabolic terms including biosynthetic process (GO:0009058), peptide biosynthetic process (GO:0043043) and amide biosynthetic process (GO:0043604) (Table S6E). This suggests D3 is a stage that readjusts its metabolism to accumulate internal energy sources. Additionally, a high representation of the term oxidoreductase activity (GO:0016491) in the C5 cluster may indicate a higher oxidative stress tolerance in D3 than other stages.

Pairwise comparison of D3 with L2, L3 and L4 identified 1036, 1792 and 1472 genes differentially expressed, respectively (Table S3). Genes up-regulated in D3 are enriched in stress response-related GO terms, including oxidoreductase activity (GO:0016491) and response to stress (GO:0006950) as well as proteolysis (GO:0006508) (Table S4). Interestingly many genes with cellulase activity (GO:0008810) are up-regulated in D3 relative to L2. Highly presented GO terms in genes down-regulated in D3 include structural constituent of cuticle (GO:0042302), proteolysis activity (GO:0006508) and lipid binding (GO:0008289) (Table S4).

Pairwise comparisons of D4 with the propagative stages identified larger numbers of DE genes than the D3 comparisons (2567, 3121 and 2618 related to L2, L3 and L4 respectively) (Table S3). Among them, numbers of D4 up-regulated genes are 1170, 1297 and 933 and GO terms related with protein kinase activity (GO:0004672), and transmembrane signalling receptor activity (GO:0004888) was significantly enriched in D4 relative to L4. GO terms enriched in genes down-regulated in D4 include structural constituent of cuticle (GO:0042302), proteolysis activity (GO:0006508) and lipid binding (GO:0008289). In particular, the GO term of aspartic-type endopeptidase activity (GO:0004190) enriched as a down-regulated function in all the three pairwise comparisons, as well as down-regulated cellulase activity (GO:0008810) relative to L4, suggests again the low feeding activity of D4 nematodes.

A direct comparison between D3 and D4 identified 1081 genes and 1033 genes up-regulated in D3 and D4, respectively (Table S4). Genes highly expressed in D3 are enriched in GO terms of proteolysis (aspartic-type endopeptidase activity (GO:0004190) and cysteine-type peptidase activity (GO:0008234)) and oxidation-reduction process (GO:0055114), whereas genes highly expressed in D4 are enriched in G-protein coupled receptor signalling pathway (GO:0007186). These results indicate substantial biological differences between the two dauer-like stages; D3 has high feeding/metabolic activity and higher stress-tolerances whereas D4 has low metabolic activity but increased and/or distinct signal pathway activity.

### Expression of genes involved in dauer pathways

To see whether nematode dauer formation/maintenance pathways are conserved in *B. xylophilus*, we compared gene expressions of *B. xylophilus* orthologues of *C. elegans* dauer genes. Four signalling pathways have major roles in dauer formation of *C. elegans*; cGMP, IIS, TGF-β, and DA/DAF-12 pathways^25–28^. *B. xylophilus* has most orthologues of *C. elegans* genes involved in these four pathways^23,29^.

**Figure 5:**
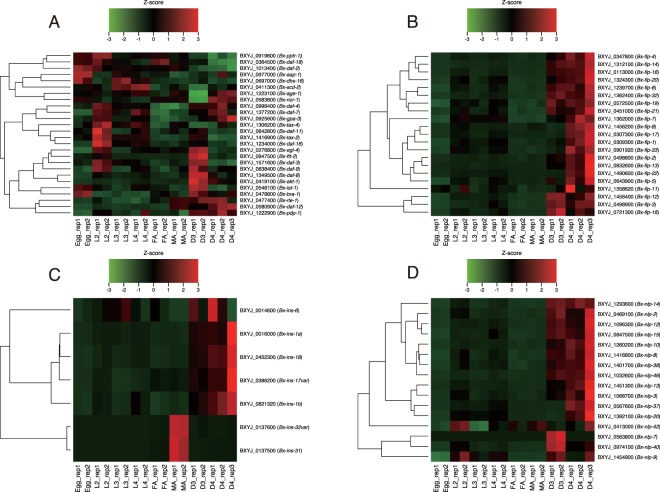
Gene expression patterns of dauer-related genes and neuropeptide genes. Heatmaps represent expression dynamics of *B. xylophilus* orthologues of (**A**) *C. elegans* dauer genes and neuropeptide (**B**) flp, (**C**) ins and (**D**) nlp genes. Rows represent genes “*B. xylophilus* gene ID (*C. elegans* gene name or *B. xylophilus* gene name)” and columns represent RNA-seq samples. The intensity of each colour denotes the standardized ratio between each value and the average expression of each gene across all samples. Genes showing null FPKM in all stages were removed from the plot.

Many *B. xylophilus* orthologues of *C. elegans* dauer genes that are up-regulated in the dauer relative to other developmental stages (Fig. S3) also exhibit high expression values in D3 and/or D4 stages (Fig. 5). Four major clusters were observed in the heatmap showing the expression pattern of the *B. xylophilus* orthologues (Fig. 5). Most orthologues of genes up-regulated in *C. elegans* dauer are included in two clusters showing high expression both in D3 and D4, particularly D3. The third cluster contained two orthologues showing high expression mainly in D4, and both *C. elegans* genes (*ncr-1* and *age-1*) showed high expression in the dauer. Orthologues in the fourth clusters have high expressions in the egg and/or L2 and relatively low in D3 and D4. Those orthologues in *C. elegans* mostly show a similar pattern (i.e. high in the egg and/or L1, and relatively low in the dauer). These results suggest that nematode dauer pathways are largely conserved between *C. elegans* and *B. xylophilus*, and *B. xylophilus* D3 and D4 use different sets of *C. elegans* dauer genes from each other to form and maintain their special developmental stages. *Bx-daf-7* (BXYJ_1377200), a single-copy orthologue of *C. elegans daf-7* which encodes a key ligand of the TGF-β signaling pathway^30^, showed a similar expression pattern to *C. elegans daf-7*, showing high expressions in the early larval stage and the dauer stages, suggesting a conserved role in the two species although diverged functions have been suggested in other nematodes^31^. There are, however, some exceptions which show a different expression pattern between *C. elegans* and *B. xylophilus*, including *tax-*4, *rle-1*, *daf-8* and *daf-16*, indicating that unique gene regulations are also present in *B. xylophilus*. Notably there are seven genes showing a null FPKM value in all stages of *B. xylophilus* (not shown in the heatmap), including BXYJ_0789700, BXYJ_1393800, and BXYJ_1599300, whose *C. elegans* homologues, *daf-38* and *pdk-1*, are active in the dauer stage.

### Expression of neuropeptide genes

Neuropeptides are short peptides acting as transmitters and neuromodulators, which can control the activity of neurons and modulate behaviour in specific way^32^. The *C. elegans* genome encodes 31 FMTFamide-like peptides (*flp* genes), 40 insulin-related peptides (*ins* genes), and 47 neuropeptide-like proteins (*nlp* genes), and they, especially *flp* genes, are significantly up-regulated in the dauer entry^33^. We identified 22 *flp*, 7 *ins* and 17 *nlp* genes in the *B. xylophilus* genome^23^. Among them 21 *flp*, 7 *ins*, and 16 *nlp* genes were expressed in at least one stage. Interestingly, we found almost all *flp* and *nlp* genes were upregulated in D3 and/or D4 (Fig. 5B,D). An exception is two *nlp* genes (*Bx*-*nlp-9* and *-42*) showing high expression in developmental stages other than D3 or D4 stage. Most of *flp* and *nlp* genes showed higher expression levels in D4 than in D3, whereas *Bx-nlp-7, nlp- 40*, *flp-3*, *flp-12* and *flp-18* showed higher expressions in D3 than in D4. The two *ins* genes (*Bx-ins-31* and *-32var*) were mainly up-regulated in adult male and the other five were up-regulated in D3 and/or D4 (Fig. 5C). In *C. elegans*, *ins-17* and *ins-18* likely work as antagonists of Daf-2 pathway, where over-expression of those ins genes leads to dauer arrest^34,35^. We found *Bx-ins-17* and *-18*, which show high expression in dauer-like stages, shares conserved cysteine bond structure with *C. elegans* though *Bx-ins-18* does not have the conserved PPG motif^36^, indicating conserved roles of those ins genes and the pathway in the two species. These results altogether suggest that those neuropeptides are tightly incorporated in the dauer biology of *B. xylophilus* as is suggested in other parasitic nematodes^33^.

### Temporal regulation of effector genes

**Figure 6:**
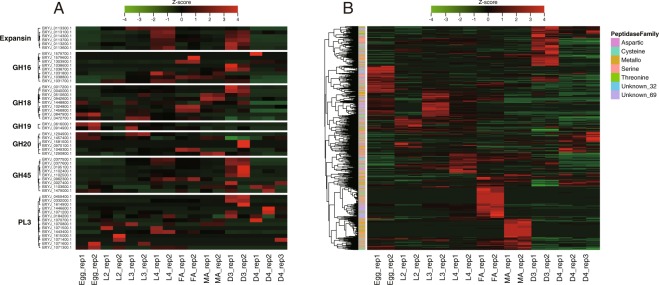
Expression dynamics of *B. xylophilus* effector genes along the development. (**A**) Heatmap of 54 genes encoding cell-wall degrading enzymes (expansin, GH45 cellulases, PL3 polysaccharide lyases, GH16 beta-1,3-glucanases, and chitin-degrading enzymes (GH18, GH19, and GH20). (**B**) Heatmap of 771 genes encoding peptidases belonging aspartic (91 genes), cysteine (139 genes), metallo (223 genes), serine (171 genes), threonine (10 genes), and unknown_69 (134 genes) families. Genes showing null FPKM in all stages were removed from the heatmap.

Plant parasitic nematodes secrete molecules affecting the host tissues called effectors^37–39^. Cell-wall degrading enzymes (CWDEs) are secreted by *B. xylophilus* and used to feed and migrate within the host tissues^23^. Expression patterns of 62 genes encoding CWDEs in *B. xylophilus* are shown in Fig. 6A, including those working on plant cell wall (GH45 cellulases, PL3 pectate lyases and expansins) and those on fungal cell wall (GH16 beta-1,3-glucanases and chitin degrading enzymes (GH18, GH19, and GH20)). Many genes both for plant and fungal CWDEs showed high expression in D3, suggesting the highly active feeding behaviour and metabolism of the dauer-like stage (please note that a sealed mouth, a typical morphological character of *C. elegans* dauers, is seen only in D4, not in D3). In particular, all highly-expressed GH45 cellulases (FPKM > 20 in any sample) showed the highest expression in D3. Additionally, D3 expansin expression is highest among the stages. It is also noteworthy that expansins and GH45 cellulases showed high FPKM values in L4 (Fig. 6A). In pectate lyases (PL3), there are three genes showing high FPKM values. Two of them (BXYJ_0459400.1 and BXYJ_0332000.1) are specifically upregulated in D3. The other PL3 gene (BXYJ_1443400.1) appears constantly expressed in all stages except eggs with the highest FPKM value in L4. In chitin degrading enzymes, GH19 genes showed high expression in eggs. Two other families (GH18 and GH20) also have a few genes that show high expression in egg, suggesting chitinase roles in egg formation or hatching. The other GH18 genes generally showed constant expression across the stages (except D4) with upregulations at D3, adult female and adult male (two genes each) (Fig. 6A). Five genes of GH16 beta-1,3-glucanases, which are involved in fungal cell wall degradation, have high expression levels at L4, adult female, and D3. Among them, two genes (BXYJ_1038600 and BXYJ_1038700) are specifically upregulated in D3 whereas the other three FPKM values are relatively constant across the stages. The D4 stage showed very low expression levels for all the enzymes with only a small number of exceptions including genes encoding chitinases, a PL3 and an expansin.

Nematode peptidases, which hydrolyse polypeptides or proteins, participate in a wide range of molecular, biological and cellular processes such as digestion of host proteins, moulting and embryonic development of the egg^40^. The *B. xylophilus* genome contains 808 peptidase genes, representing the highest gene number among characterised nematode genomes (Table S8)^23^, which are composed of aspartic (106 genes), metallo (230 genes), cysteine (142 genes), serine (170 genes), threonine (13 genes), unknown_32 (8 genes) and unknown_69 (136 genes). Gene expression pattern of the peptidases is visualised in Figs 6B and S4. Intriguingly, most peptidases specifically upregulate at only one developmental stage comprising clear stage-specific clusters in the heatmap. The clusters have a similar size to each other except L2- and D4-specific clusters which show fewer genes. Each stage-specific cluster contains various types of peptidase (i.e. aspartic, cysteine, metallo, serine, threonine peptidase, unknown_32 and unknown_69 peptidases). The number of genes expressed in each life stage of *B. xylophilus* varies depending on peptidase type. The female-specific cluster has more highly expressed genes of unknown_69 type and the D3 cluster has more highly expressed genes of aspartic, cysteine, serine and threonine types. In contrast, the number of highly expressed genes of metallo and unknown_32 type peptidase families is increased in L3- and male-specific clusters (Figs 6B and S4).

## Discussion

In the present study, we performed a comprehensive stage-specific transcriptome analysis of *B. xylophilus* and identified genes differentially expressed between developmental stages.

*B. xylophilus* is a migratory endoparasite which multiplies within the host via multiple generations unlike other major plant parasitic nematodes, including root-knot and cyst nematodes, which are sedentary parasites whose developmental stages have clear stage-specific roles in their lifecycles^3,41^. This indicates that all propagative stages of *B. xylophilus* share the same niche in the host and therefore, previous studies paid little attention to stage differences within the propagative life cycle. When investigating the pathogenicity of *B. xylophilus*, many studies used mixed-stage nematodes^2,11,42–44^ though differences between the dispersal and propagative stages have been more extensively studied^7,45–47^. Recently, two independent transcriptome studies investigated gene expression of *B. xylophilus* in the host plants and identified ‘effectors’ and genes important for parasitism^20,22^. However, those studies also used mixed-stage nematodes and did not reveal roles in the pathogenic life cycle or particular biological characteristics of each developmental stage.

In this study, we found that many genes are differentially regulated in *B. xylophilus* even between the propagative stages. For example, genes involved in germline development in *C. elegans* were up-regulated in the adult stages of *B. xylophilus*: genes with GO terms of protein kinase and phosphatase in males, and those with GO terms of oocyte maturation in females. Genes involved in DNA replication and pharynx development were up-regulated in eggs. In addition, genes involved in moulting such as collagen genes are likely to have oscillating expression patterns within each larval stage as seen in *C. elegans*^48^. These results suggest that roles and regulation of genes involved in basic biological processes are well conserved between *B. xylophilus* and *C. elegans* though the two species belong to different evolutionary clades from each other^21^.

Intriguingly we also found that genes encoding effectors involved in parasitism are differentially expressed between stages. In particular, genes encoding CWDEs were highly expressed in D3 and L4 relative to other propagative stages indicating their highly active feeding status. This may reflect a high demand for nutrients by D3 to facilitate lipid granule accumulation in the body for long-term survival, or by L4 to develop the germlines. It also indicates those stages may have particular roles in the pathogenesis of the nematode by destroying plant tissues.

Genes encoding peptidases, which are highly expanded in the *B. xylophilus* genome^23^, were also differentially expressed between stages. Within a peptidase family, *B. xylophilus* is likely to use different sets of genes from the multiple gene copies depending on the stage. It is known that peptidase families have a diverse range of biological roles such as moulting, development, food digestion, and parasitism in nematodes. Our results suggest that each gene in an expanded peptidase family has distinct roles or distinct regulation in *B. xylophilus*, representing a clear example of gene family evolution by gene duplication and functional divergence. Stage-specific regulation of genes in an expanded gene family were also observed in other parasitic nematodes such as SCP/TAPS gene families in animal parasitic *Strongyloides* species^49^ and *Necator* species^50^, astacin-like metallopeptidase in *Strongyloides* species^49^, the cysteine peptidase gene family in *Schistosoma mansoni*^51^, ShKT-domain containing proteins in *Teladorsagia circumcincta*^52,53^, and metallopeptidases in *Globodera pallida*^54^.

Another example of genome evolution via gene duplication and functional divergence are the chitinase gene families. *B. xylophilus* has a higher number of GH18 chitinase genes than other obligate plant parasites^23^ and those genes are likely to have different roles in *B. xylophilus*. Expression of nine GH18 chitinase genes in *B. xylophilus* showed variable patterns (Fig. 6A). This result is mostly consistent with a previous study^55^, where they cloned seven chitinase genes in *B. xylophilus* designated *Bx-Chi-1* to *Bx-Chi-7* and performed functional analysis. They grouped *B. xylophilus* chitinase genes into four types: egg-specific involved in egg hatching (*Bx-chi-7*), female spermatheca-specific involved in reproduction in females (*Bx-chi-1*), male-specific involved in sperm metabolism (*Bx-chi-3, 4, 5* and *6*), and pharyngeal gland-specific involved in fungal feeding (*Bx-chi-2*). In this study, we found that two genes including BXYJ_1024800.1 and BXYJ_1456800.1 were highly expressed in the adult female stage and one gene (BXYJ_0847900.1) was observed to be highly expressed in eggs. Three chitinase genes including *Bx-chi-3* and *4* (BXYJ_1448800.1 and BXYJ_0510600.1) were mainly up-regulated in adult males. Two genes, including *Bx-chi-2* (BXYJ_0040000.1), were highly expressed in larval stages with the highest expression at D3, likely involved in fungal feeding. These results support the idea that each of multiple GH18 chitinases in the genome has a distinct role in its lifecycle^55^, from general nematode biology including reproduction and development to a specific fungal feeding character of *B. xylophilus*.

*C. elegans* dauer is a specialised stage favouring survival and dispersal whose characteristics include tolerance to starvation and high stress^56^. Parasitic nematodes also have the same type of arrested stage, called “infective juveniles (or infective larvae)” which are normally an alternative 3^rd^ stage larva and can survive harsh conditions while searching for a new host^57^. Unlike many other nematodes, *B. xylophilus* possesses two developmental stages with dauer-like characteristics in its life cycle (D3 and D4). D3 emerges in the host tree when the host is dying (harsh conditions). D4 larvae develop from D3 and are dispersed by the vector beetle. We found that the two dauer-like stages have very different gene expressions not only from other propagative stages, but also between the two stages. Many CWDEs and peptidases involved in “food degradation” were highly up-regulated in D3 relative to the propagative stages, whereas D4 showed a low metabolic-state gene expression pattern like the *C. elegans* dauer^58^. D3 doesn’t have a typical morphological character of dauers - a sealed buccal opening, indicating D3’s active feeding state, whereas D4 has a sealed mouth and degenerate digestive systems, showing no-feeding behavior^7^. In this sense, D3 is more like *C. elegans* pre-dauer (or L2d) which is the alternative 2^nd^ stage larva *C. elegans* forms under harsh conditions before progressing onto dauer arrest^59^. However, the stress tolerance and longevity of D3 are similar to those of the *C. elegans* dauer. Genes involved in stress responses, including those encoding oxidoreductase and deubiquitinating enzymes, were more active in D3 than D4, which reflects the natural life cycle where D3 survives in the host tree after emergence from autumn till spring, suggesting its long life, which is another typical character of the *C. elegans* dauer and infective larvae of other parasitic nematodes. In contrast, *B. xylophilus* D4 occurs only at the presence of the vector beetle and is likely specialized for dispersion. Genes involved in signalling pathways were up-regulated in D4. This is probably because D4 needs more receptors and signal mediators to successfully recognize the insect vector. These results suggest that *C. elegans* dauer abilities and roles are apportioned between the two *B. xylophilus* dauer-like stages to achieve the unique *B. xylophilus* life cycle.

The possession of two dauer-like stages of *B. xylophilus* is uncommon in nematodes. Even within the genus, only a few species have this two-step dauer formation^60^. This atypical dauer formation seem adaptive to its life history, *i.e*., the worms propagate in the dead/dying wood, and transmitted by longhorn beetle species. The vector longhorn beetles, *Monochamus* spp. (and their relatives associated with other *Bursaphelenchus* spp.) are univoltine^61^, and can carry the nematodes only in early summer season, *i.e*., *B. xylophilus* which is highly specialized to its vector insects has to survive from autumn to next early summer under harsh conditions.

Several signalling pathways including cGMP^62^, insulin-like^26^, TGF-β^25^ and DA/DAF-12^27^ control *C. elegans* dauer formation. We found that orthologues of genes involved in those pathways as well as genes encoding neuropeptides were also present in *B. xylophilus*. Among them, genes upregulated in *C. elegans* dauer showed high expression values in D3 and/or D4 in *B. xylophilus*. In particular, *B. xylophilus* orthologue of *daf-12* (BXYJ_0580900), a key regulator of DA/DAF-12 pathways in *C. elegans*^25^, is up-regulated in D4 of *B. xylophilus*, suggesting the pathways are conserved in *B. xylophilus* as in some other nematodes^63–65^. Orthologues of *egl-4* (BXYJ_0276800) in cGMP pathway associated with receiving chemical signals, and *ist-1* (BXYJ_0518100 and BXYJ_0548100) and *akt-1* (BXYJ_0419100) in IIS pathway associated with transmission of signals to downstream pathways showed high expression pattern in D3 as in the *C. elegans* dauer, but not in D4. This again indicated that D3 and D4 of *B. xylophilus* are similar to *C. elegans* dauer but have distinct characteristics from each other. However, we should also note that post-translational regulations should play important roles in those pathways and may be different between species. It may be noteworthy that expression of some dauer pathway orthologues including orthologue of *daf-38* were not detected in any stages of *B. xylophilus* in this study. *C. elegans daf-38* encodes a G-protein-coupled receptor which binds an ascaroside, dauer-inducing pheromone^66^, indicating diverged environmental signal receptions between these two species although ascarosides are an evolutionarily conserved family of nematode pheromones and ascaroside-like substances with heat-tolerant and water-soluble characteristics seem to be used by *B. xylophilus* to induce D3 formation^12^.

FMRFamide-like peptide genes (*flp*) are specifically upregulated during dauer entry in *C. elegans* and are shown to be involved in the dauer specific nictation behaviour and CO_2_ attraction^33^. This seems to be conserved in many parasitic nematodes, whose infective juveniles show similar behaviours^33^. We observed upregulation of *flp* genes in D3 and D4 of *B. xylophilus*. This is interesting because strong nictation behaviour was seen only in D4 in *B. xylophilus* though both D3 and D4 are attracted by CO_2_ ^4^. The *flp* genes that were differentially expressed between D3 and D4 may be responsible to those differences.

Finally, the results and data from this study provide a foundation for further investigation of *B. xylophilus* biology and the development of novel pathogen control strategies. *B. xylophilus* transcriptome data and unique life cycle also provide a unique opportunity to perform comparative studies on many conserved and not-conserved processes for which the free-living *C. elegans* remains the pre-eminent model.

## Methods

### Genome reference

Illumina reads from 600 bp paired-end and 3-kb mate-pair libraries, generated using DNA extracted from the *B. xylophilus* Ka4C1 isogenic line^23^, were assembled using the MaSuRCA assembler 2.2.1^67^ with parameters (filter 60X- with cgwErrorRate = 0.2). The resulting assembly was further improved by the Gapfiller^68^ and Image^69^ to fill gaps in the initial assembly using Illumina pair-end reads. After base corrections using ICORN^70^, haplotypes within the assembly were collapsed using Haplomerger2^71^.

For the gene prediction, Augustus (v. 3.0.1)^72^ was trained for *B. xylophilus* based on a training set of about 500 confident genes, manually curated in Artemis^73^ using aligned RNA-seq data and transfers of the previous version of gene models (v1.2)^23^. RNA-seq alignments to the genome using TopHat v.2.0.11^74^ were used to produce intron hints. Cufflinks (v2.0.1)^75^ was used to assemble transcript fragments, and this information was converted into exon hints. The trained versions of Augustus were run using all the hints for that species as input to generate v2.0 gene models. Functional annotations and Gene Ontology (GO) term assignments were performed using Pfam scan (v27.0)^76^, Interproscan^77^ and Blast2Go v2^78^.

### RNA sequencing

Nematodes (*B. xylophilus* strain Ka4C1) were cultured on a fungal mat of *Botrytis cinerea* on PDA (39 g/L Potato dextrose agar, Nissui) plate for 4 days and eggs were collected as described in Iwahori and Futai^79^. Collected eggs were incubated in distilled water at 25 °C for 28 h to let them hatch and age-synchronised. Age-synchronised L2s were then transferred to a 1/4 PDA (4% agar) plate with *B. cinerea* inoculated a priori and cultured at 25 °C. L2, L3, and L4 nematodes were harvested at 3 h, 20 h, and 38 h post nematode inoculation, respectively. Adult males and females were obtained from 52 h culture by hand-picking using a needle. D3 nematodes were collected from two-month old cultures (1/5 PDA supplemented with 3% glycerol). Nematodes of D4 stage were produced and isolated using the artificial pupal chamber method^18,80^. Nematodes were frozen in 150 µL TRI reagent (Life Technology) in liquid nitrogen and homogenised using Biomasher (TaKaRa). This step was repeated >6 times till all nematode bodies were disrupted. Total RNA was then extracted according to TRI reagent standard procedures (Life Technology).

Total RNA samples were qualified using the Bioanalyzer 2100 (Agilent Technology, Inc.). Only samples with an RNA integrity value (RIN) greater than 7.0 were used for library construction. One hundred ng of total RNA was used to construct an Illumina sequencing library using the TruSeq RNA-seq Sample Prep kit according to the manufacturer’s recommended protocols (Illumina, San Diego, USA). The libraries were sequenced for 101-bp paired-ends on an Illumina HiSeq2000 sequencer using the standard protocol (Illumina). RNA-seq experiments were conducted in triplicate for D4 and in duplicates for the other stages.

### Differential gene expression analysis

RNA-seq reads were mapped against the *B. xylophilus* genome reference (v2.0) using Tophat v.2.0.^74^ under options (fr-unstranded, minimum intron length of 15, maximum intron length of 50,000, microexon search; mate inner distance of 100, mate standard deviation of 40). Host RNA contaminations were checked by mapping RNA-seq reads to the *M. alternatus* reference genome (T. Kikuchi, unpublished) using Tophat v.2.0 with aforementioned options. Mapped read count of each gene was calculated using HTSeq with options (−s no, −a 10, −m union)^81^ and differential expression analyses were performed using EdgeR v3.2.4^82^. A transcript was identified as differentially expressed in a pairwise comparison if the following criteria were met: false discovery rate (FDR) ≤0.001 and fold change ≥2.0. FPKM values were calculated using Cufflinks packages v2.2.1^75^ and used to generate for MDS plot using “cmdscale” and ggplot2^83^ and for correlation coefficient heatmap using “cor” and “heatmap.3” functions implemented in R (version 3.1.3)^84^. Gene expression heatmaps were generated by converting the FPKM values to z- scores with clustering per gene based on Ward’s hierarchical agglomerative clustering method (Euclidean distance measure; Ward.2 criterion) using ComplexHeatmap R Bioconductor package^85^.

We used the method described by Choi *et al*.^24^ to elucidate somatic-enriched and germline-enriched genes defined as:

$$\begin{array}{rcl}{\rm{somatic}}-{\rm{enriched}} & = & ({\rm{genes}}\,{\rm{upregulated}}\,{\rm{in}}\,{\rm{adult}}\,{\rm{female}}\,{\rm{relative}}\,{\rm{to}}\,{\rm{eggs}})\\ {\rm{germline}}-{\rm{enriched}} & = & (\mathrm{genes}\,{\rm{upregulated}}\,{\rm{in}}\,{\rm{eggs}}\,{\rm{relative}}\,{\rm{to}}\,\mathrm{male})\,{\rm{or}}\\  &  & ({\rm{genes}}\,{\rm{upregulated}}\,{\rm{in}}\,{\rm{eggs}}\,{\rm{and}}/{\rm{or}}\,{\rm{female}}\,{\rm{relative}}\,{\rm{to}}\,{\rm{other}}\,{\rm{stages}})\\  &  & -({\rm{somatic}}-{\rm{enriched}}\,\mathrm{genes}){\rm{.}}\end{array}$$

Temporal gene expression patterns were clustered by the k-means method using the STEM program^86^. The optimal cluster number k = 10 was estimated by the gap statistic method^87^ using R (v3.3.3)^84^ and the factoextra package. Gene ontology term enrichment analyses were performed using the GOseq (v1.4.0)^88^ package implemented in R program (v3.1.3)^84^ or the function implemented in STEM^86^.

### Orthologue analyses

Based on stage specific RNA-seq analyses^48^, *C. elegans* genes were categorized into three groups; ‘flat’ showing stable expression in propagative juveniles, ‘rising’ showing increasing expression along the nematode’s growth and ‘oscillating’ showing pervasive and phase-locked oscillations of gene expression in developing juveniles. The oscillatory pattern demonstrates an eight-hour periodic cosine-curve wave. Therefore, when comparing gene expression between stages, those genes may lead to false positives^43^. Orthologous relationships between *B. xylophilus* and *C. elegans* genes were identified using OrthoMCL version 5^89^. The orthologue information (orthofamily) was used to classify *B. xylophilus* genes into the three expression pattern categories (‘flat’, ‘rising’ and ‘oscillation’) according to Hendirks *et al*.^48^.

### Identification of effector candidate genes

Genes encoding cell wall degrading enzymes and expansins in the v2.0 genome were identified using dbCAN^90^ and BLASTP^91^. Peptidases were identified using MEROPS v.11.0^92^ with HmmerWeb v.2.19 search engine^93^ (with cut-off e-value 0.01). Peptidases were classified into aspartic, cysteine, metallo, serine, threonine, unknown_32 (collagenase) and unknown_69 (self-processing peptidase) types. Non-peptidase homologues and peptidase inhibitors were not considered in this study.

### Identification of dauer genes and neuropeptide genes

OrthoMCL results (please see above) were used to identify orthologous genes of *C. elegans* dauer genes^29^ and neuropeptide genes in *B. xylophilus*. The orthologous relationships were confirmed using the orthologue gene trees in the Wormbase Parasites^94^. Gene expressions of *C. elegans* genes in each developmental stage were derived from Wormbase WS220 (http://www.wormbase.org; expression page)^95,96^.

## Supplementary information

Supplementary Figs and Tables

Supplementary Table S6

Supplementary Table S7

Supplementary Table S4

## Data Availability

The genome assembly/annotation and RNA sequence data has been deposited to NCBI/EMBL/DDBJ under BioProject accession ID PRJDB7519 and PRJDB3458, respectively.
