# Supplementary material for: Stage-specific transcriptome of Bursaphelenchus xylophilus reveals temporal regulation of effector genes and roles of the dauer-like stages in the lifecycle
Source: Sci Rep. 2019 Apr 15;9:6080. doi: 10.1038/s41598-019-42570-7 (PMC6465311; doi:10.1038/s41598-019-42570-7)
Supplement: Supplementary file 1 — Supplementary Figs and Tables [file 41598_2019_42570_MOESM1_ESM.pdf]

Supplementary information for

# Stage-specific transcriptome of *Bursaphelenchus xylophilus* reveals temporal regulation of effector genes and roles of the dauer-like stages in the lifecycle

Suguru E. Tanaka[1,+], Mehmet Dayi[2,3,+], Yasunobu Maeda[2], Isheng J Tsai[4], Ryusei Tanaka[2], Mark Bligh[2], Yuko Takeuchi-Kaneko[5], Kenji Fukuda[1], Natsumi Kanzaki[6], and Taisei Kikuchi[2]*

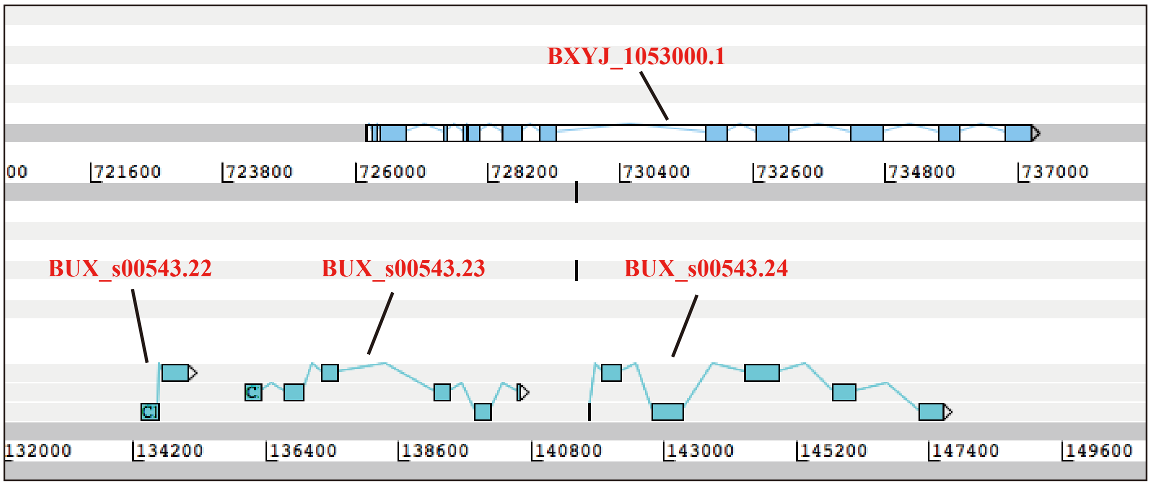


Figure S1. An example of the new (V2.0) and corresponding old (V1.2) gene models. Three old gene models (BUX_s00543.22, 225 aa, BUX_s00543.23, 427 aa and BUX_s00543.24, 686 aa, respectively) were connected into one (BXYJ_1053000.1, 1107 amino acids) in the new gene set.

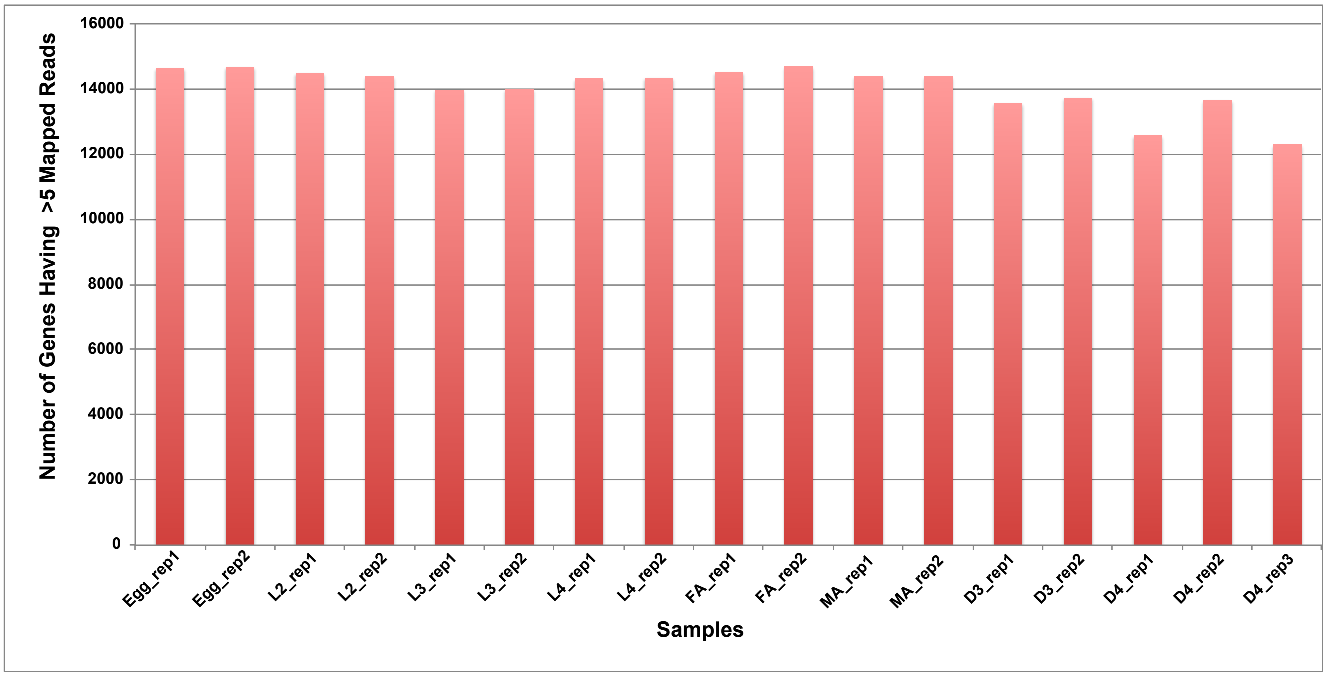


Figure S2. Number of B. xylophilus genes that have over 5 mapped reads in each sample. Each sample has more than 12000 those genes.

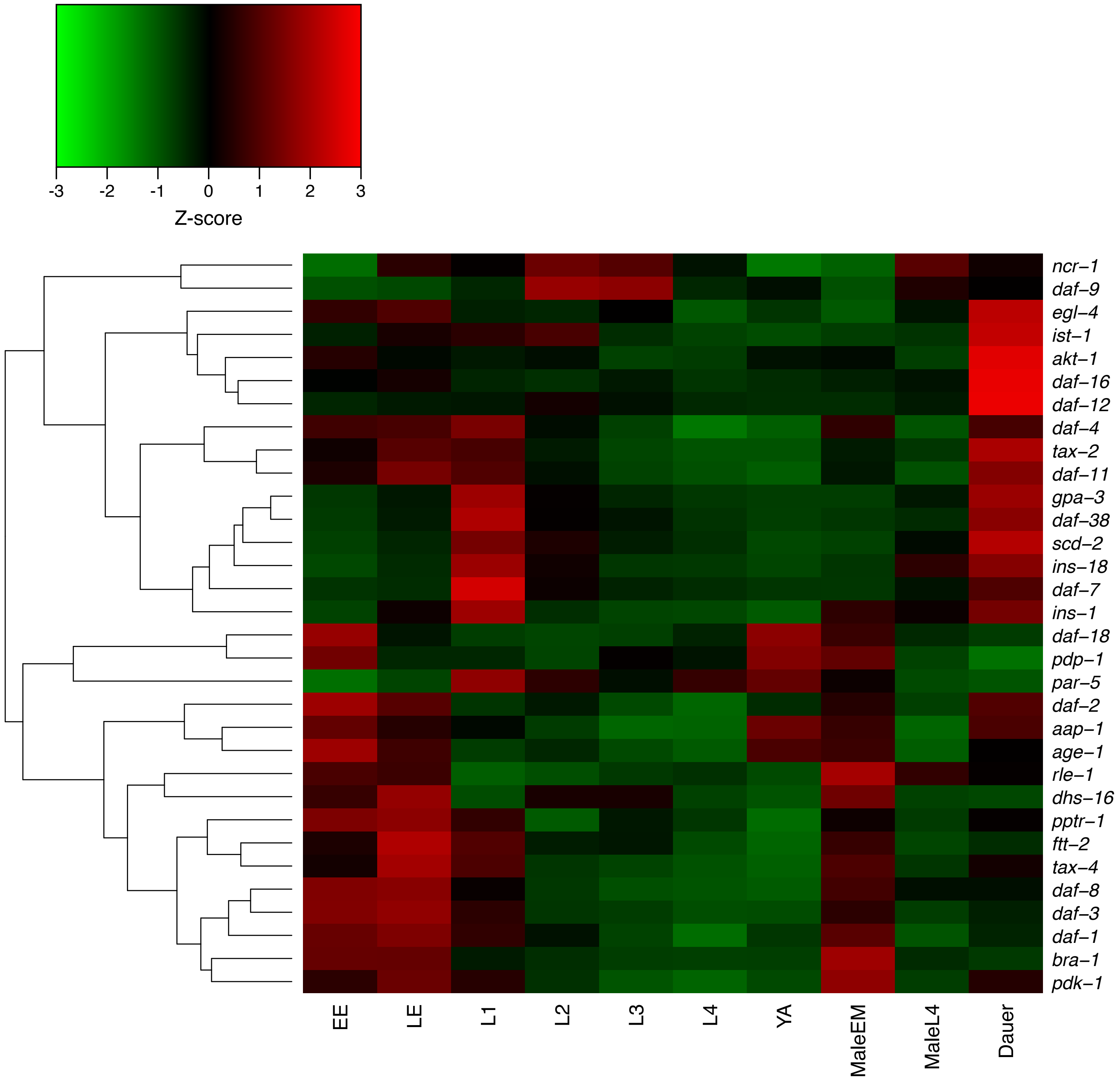


Figure S3. Heatmap showing expression of dauer genes in C. elegans stages. The data was derived from the WormBase (http://www.wormbase.org).

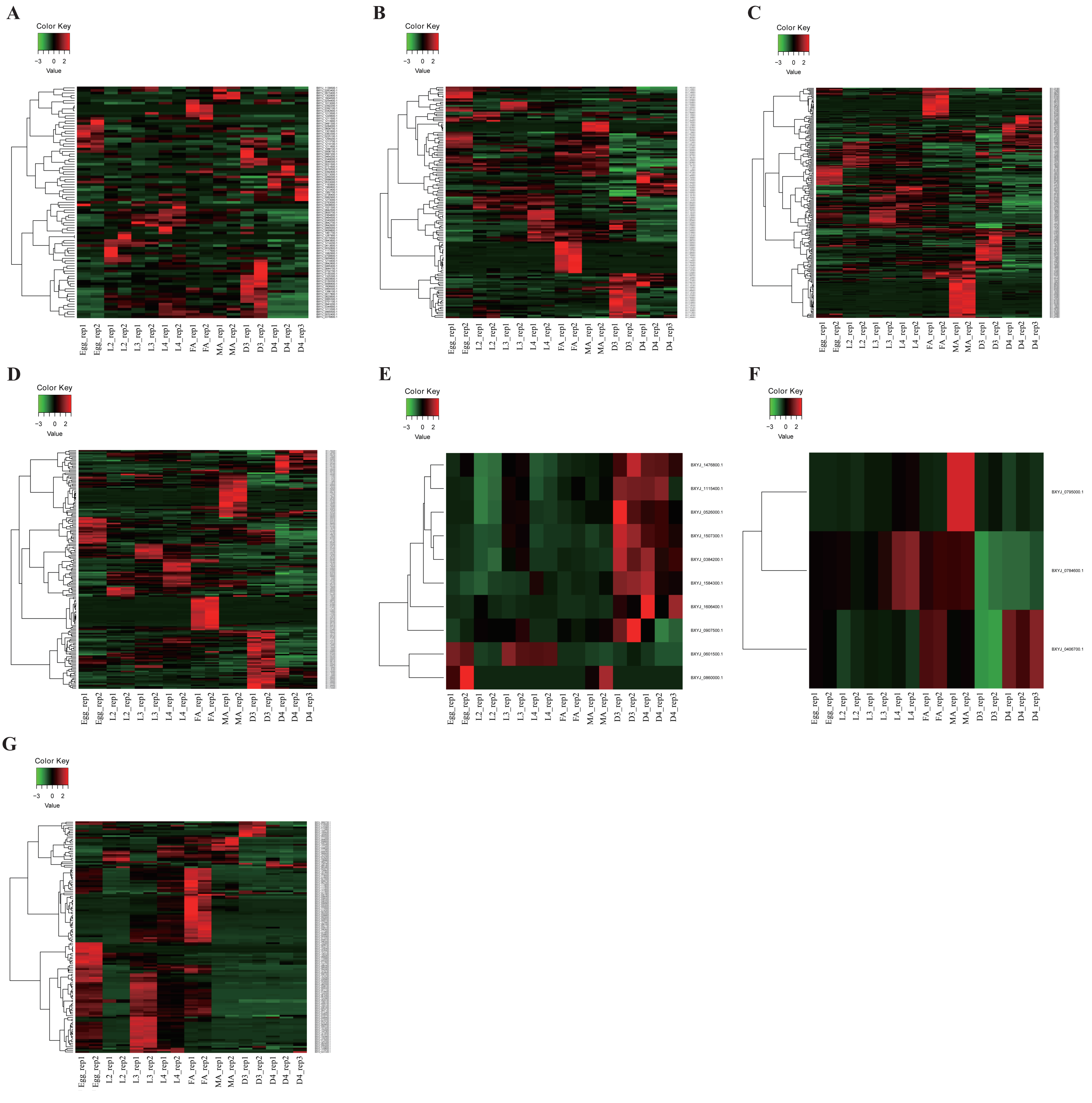


Figure S4. Expression dynamics of peptidase genes in B. xylophilus developmental stages. Heatmaps were generated for each peptidase family; (A) aspartic (91 genes), (B) cysteine (139 genes), (C) metallo (223 genes), (D) serine (171 genes), (E) threonine (10 genes), (F) unknown_32 (3 genes), and (G) unknown_69 (134. genes).

**Table S1.** Genome and gene model stats for the old (v1.2) and new (v2.0) assemblies.

| | Genome version | |
|---|---|---|
| | v.1.2 | v.2.0 |
| Total size of assemblied sequence (Mb) | 74.6 | 75.9 |
| Number of scaffolds | 1,231 | 501 |
| N50 of scaffolds (kb) | 1,158 | 1,577 |
| Maximum length of scaffold (kb) | 3,612 | 4,119 |
| Gap length (kb) | 1,475 | 42 |
| GC content (%) | 40.4 | 40.4 |
| Completeness by CEGMA (%): (complete/partial) | 97/98 | 98/98 |
| Completeness by BUSCO (%) | 78 | 79 |
| Average CEGMA gene number: (complete/partial) | 1.08/1.09 | 1.18/1.23 |
| Mapped read ratio (%) | 98.25 | 99.09 |
| Proper-pair ratio (%) | 91.64 | 96.4 |
| Number of gene models | 18,074 | 16,346 |
| Number of Genes | 18,074 | 16,346 |
| Number of Transcript | 18,074 | 16,346 |
| Average Transcript Length (bp) | 1,572 | 2,735 |
| Median Transcript Length (bp) | 1,214 | 1,811 |
| Total Transcript Length (bp) | 28,429,143 | 44,709,149 |
| Average Exons per gene | 4.51 | 5.89 |
| Median Exons per gene | 4 | 5 |
| Number of Exon | 81,543 | 96,239 |
| Average Exon Length (bp) | 228.88 | 215.21 |
| Median Exon Length (bp) | 183 | 172 |
| Total Exon Length (bp) | 18,663,247 | 20,711,270 |
| Number of Intron | 63,461 | 79,892 |
| Average Intron Length (bp) | 153.02 | 300.33 |
| Median Intron Length (bp) | 69 | 77 |
| Total Intron Length (bp) | 9,710,974 | 23,994,071 |

**Table S2.** (in a separate file) RNA-seq reads and mapping stats and FPKM (Fragments Per Kilobase of transcript per Million mapped reads) values of B. xylophilus genes in life stages

**Table S3.** Number of differentially expressed genes in pair-wise comparisons.

| Stage | Egg | L2 | L3 | L4 | FA | MA | D3 | D4 |
|---|---|---|---|---|---|---|---|---|
| **Egg** | - | - | - | - | - | - | - | - |
| **L2** | 2982 | - | - | - | - | - | - | - |
| **L3** | 1641 | 1232 | - | - | - | - | - | - |
| **L4** | 2316 | 1088 | 347 | - | - | - | - | - |
| **FA** | 2606 | 2764 | 1852 | 1367 | - | - | - | - |
| **MA** | 4327 | 3339 | 3502 | 3191 | 2653 | - | - | - |
| **D3** | 3398 | 1036 | 1792 | 1472 | 2114 | 2895 | - | - |
| **D4** | 4260 | 2567 | 3121 | 2618 | 3172 | 4141 | 2112 | - |

**Table S4.** (in a separate file) Gene Ontology enrichment of differentially expressed genes in pair-wise comparisons.

**Table S5**. (in a separate file) Number of DE genes in life stages comparisons and assignment as “flat”, “rising” (rises over time), “oscillating” and “others”.

| Pairwise | Flat | Rising | Oscillating | Others | Total |
|---|---|---|---|---|---|
| Egg vs L2 | 431(14.5) | 33(1.1) | 365(12.2) | 2153(72.2) | 2982 |
| Egg vs L3 | 210(12.8) | 27(1.6) | 74(4.5) | 1330(81.0) | 1641 |
| Egg vs L4 | 395(17.1) | 35(1.5) | 127(5.5) | 1759(75.9) | 2316 |
| Egg vs FA | 359(13.8) | 67(2.6) | 225(8.6) | 1955(75.0) | 2606 |
| Egg vs MA | 476(11.0) | 152(3.5) | 410(9.5) | 3289(76.0) | 4327 |
| Egg vs D3 | 612(18.0) | 45(1.3) | 433(12.7) | 2308(67.9) | 3398 |
| Egg vs D4 | 904(21.2) | 73(1.7) | 404(9.5) | 2879(67.6) | 4260 |
| L2 vs L3 | 91(7.4) | 11(0.9) | 320(26.0) | 810(65.7) | 1232 |
| L2 vs L4 | 82(7.5) | 14(1.3) | 240(22.1) | 752(69.1) | 1088 |
| L2 vs FA | 487(17.6) | 73(2.6) | 214(7.7) | 1990(72.0) | 2764 |
| L2 vs MA | 405(12.1) | 156(4.7) | 122(3.7) | 2656(79.5) | 3339 |
| L2 vs D3 | 182(17.6) | 12(1.2) | 98(9.5) | 744(71.8) | 1036 |
| L2 vs D4 | 514(20.0) | 68(2.6) | 149(5.8) | 1836(71.5) | 2567 |
| L3 vs L4 | 18(5.2) | 6(1.7) | 69(19.9) | 254(73.2) | 347 |
| L3 vs FA | 150(8.1) | 70(3.8) | 217(11.7) | 1415(76.4) | 1852 |
| L3 vs MA | 261(7.5) | 155(4.4) | 377(10.8) | 2709(77.4) | 3502 |
| L3 vs D3 | 186(10.4) | 22(1.2) | 375(20.9) | 1209(67.5) | 1792 |
| L3 vs D4 | 442(14.2) | 79(2.5) | 384(12.3) | 2216(71.0) | 3121 |
| L4 vs FA | 140(10.2) | 61(4.5) | 63(4.6) | 1103(80.7) | 1367 |
| L4 vs MA | 198(6.2) | 152(4.8) | 289(9.1) | 2552(80.0) | 3191 |
| L4 vs D3 | 146(9.9) | 13(0.9) | 300(20.4) | 1013(68.8) | 1472 |
| L4 vs D4 | 303(11.6) | 62(2.4) | 310(11.8) | 1943(74.2) | 2618 |
| FA vs MA | 102(3.8) | 149(5.6) | 229(8.6) | 2173(81.9) | 2653 |
| FA vs D3 | 170(8.0) | 54(2.6) | 267(12.6) | 1623(76.8) | 2114 |
| FA vs D4 | 526(16.6) | 56(1.8) | 281(8.9) | 2309(72.8) | 3172 |
| MA vs D3 | 203(7.0) | 153(5.3) | 106(3.7) | 2433(84.0) | 2895 |
| MA vs D4 | 588(14.2) | 158(3.8) | 190(4.6) | 3205(77.4) | 4141 |
| D3 vs D4 | 396(18.8) | 46(2.2) | 138(6.5) | 1532(72.5) | 2112 |
| non-DEgene* | 3051(45.3) | 24(0.4) | 207(3.1) | 3452(51.3) | 6734 |

*Genes that are not differentially expressed in any pairwise comparisons. Numbers in parentheses are percentage of the total.

**Table S6.** (in a separate file) Gene Ontology (GO) enrichment results for genes clustered along development. GO analysis of (A) C1, (B) C2, (C) C3, (D) C4, (E) C5, (F) C6, (G) C7, (H) C8, (I) C9, and (J) C10 cluster.

**Table S7**. (in a separate file) Gene Ontology enrichment results of germline (A)- and somatic-enriched (B) genes.

**Table S8.** Comparison of numbers of peptidases in genomes of representative nematode species.

| Species[1] | Clade[2] | Clade[3] | lifestyle | Number of peptidases in genome[4] |
|---|---|---|---|---|
| *Bursaphelenchus xylophilus* | IV | 10 | plant parasitic | 808 |
| *Meloidogyne incognita* | IV | 12 | plant parasitic | 124 |
| *Globodera pallida* | IV | 12 | plant parasitic | 236 |
| *Panagrellus redivivus* | IV | 10 | free-living | 416 |
| *Caernohabditis elegans* | V | 9 | free-living | 435 |
| *Brugia malayi* | III | 8 | animal parasitic | 402 |
| *Strongyloides stercoralis* | IV | 10 | animal parasitic | 439 |
| *Ascaris suum* | III | 8 | animal parasitic | 337 |
| *Necator americanus* | V | 9 | animal parasitic | 271 |
| *Romanomermis culicivorax* | I | 2 | entomoparasitic | 218 |

1 Genome sequences were retrived from wormbase release WS266
2 Blaxter ML et al. (1998) Nature 392:71–75
3 van Megen H et al. (2009) Nematology 11:927–50
4 Peptidases were identified by MEROPS Blast (version 11.0)
